# Interplay Between Enteroendocrine Hormone (Leptin) and Adipokines (Ghrelin and Adiponectin) with Gastric Expression of *FTO* and *MC4R* Genes

**DOI:** 10.1038/s41598-025-29899-y

**Published:** 2025-12-21

**Authors:** Mohamed Hany, Mona K. ElDeeb, Ehab Elmongui, Anwar Ashraf Abouelnasr, Noha A. El-Banna, Sahar M. Omer, Sara A. Shaker, Rasha A. ElTahan

**Affiliations:** 1 Bariatric and Metabolic Surgery, Madina Women’s Hospital, Alexandria, Egypt; 2 Clinical Laboratory Sciences, Jouf University, Sakaka, Saudi Arabia; 3 Chemical Pathology Department, Medical Research Institute, Alexandria University, Alexandria, Egypt; 4 Independent Biostatistical Consultant, Alexandria, Egypt; 5 Biochemistry Department, Medical Research Institute, Alexandria University, Alexandria, Egypt; 6 Department of Surgery, Medical Research Institute, Alexandria University, Alexandria, Egypt

**Keywords:** Obesity, FTO gene, MC4R gene, Adipokines, Leptin, Gastric gene expression, Genetics, Medical research

## Abstract

Obesity is a complex, multifactorial disease influenced by genetic, hormonal, and metabolic factors. The fat mass and obesity-associated (FTO) and melanocortin 4 receptor (MC4R) genes have been implicated in body weight regulation through gut–brain signaling and their interactions with adipokines and enteroendocrine hormones. This study investigated the association between gastric expression of FTO and MC4R genes and circulating levels of leptin, adiponectin, and ghrelin in individuals with and without obesity. We conducted a case–control study including 50 patients with obesity undergoing sleeve gastrectomy and 50 controls undergoing diagnostic endoscopy. Gastric tissue gene expression was assessed by qRT-PCR, and serum hormone levels were quantified using ELISA. Inverse propensity score weighting was used to adjust for age and sex. FTO expression was significantly upregulated in patients with obesity (fold-change: 5.8 vs. 1.0, *p* < 0.001), showing a parallel elevation with adiponectin and BMI at the group level. In contrast, MC4R expression was significantly downregulated (fold-change: 0.1 vs. 1.0, *p* < 0.001), and positively associated with HOMA-IR and showed a borderline positive trend with fasting blood glucose (*p* = 0.074). Adiponectin levels were paradoxically elevated in the obesity group and correlated with both BMI and HDL. Leptin and ghrelin levels showed no significant group differences. These findings suggest that altered gastric expression of FTO and MC4R may contribute to obesity-related metabolic disturbances through peripheral adipokine pathways. Further investigation into tissue-specific gene–hormone interactions may inform novel therapeutic strategies for obesity.

## Introduction

Obesity is a complex and multifactorial disease characterized by excessive adiposity and associated with increased risks of cardiovascular disease, type 2 diabetes mellitus, and various cancers^1^. Its pathogenesis involves intricate interactions among genetic, hormonal, metabolic, and environmental factors^1–4^. In recent years, focus has expanded beyond central regulatory mechanisms to include peripheral contributors, including adipose tissue-derived adipokines and enteroendocrine hormones of the gastrointestinal tract^5–8^.

Leptin, adiponectin, and ghrelin are key hormones implicated in the regulation of appetite, energy homeostasis, and insulin sensitivity. Leptin, primarily produced by adipose tissue, suppresses appetite via hypothalamic melanocortin pathways involving the melanocortin 4 receptor (MC4R)^9^. Adiponectin is known for its insulin-sensitizing, anti-inflammatory, and cardioprotective properties^10^. Ghrelin, an orexigenic hormone secreted by the stomach, stimulates appetite and promotes weight gain^11^. Altered levels of these hormones are frequently observed in individuals with obesity and contribute to the development of metabolic dysfunction^7^.

At the genetic level, the fat mass and obesity-associated gene (FTO) and the MC4R gene are among the most consistently linked with obesity through genome-wide association studies^12–14^. While these genes have been extensively studied in the context of central nervous system pathways and systemic polymorphisms^15–17^, their expression in peripheral tissues such as the stomach remains poorly understood.

This study aimed to investigate the gastric expression of FTO and MC4R genes and their association with circulating levels of leptin, adiponectin, and ghrelin in individuals with and without obesity. By exploring this tissue-specific relationship, we sought to clarify whether gut-localized gene expression contributes to systemic hormonal regulation and the metabolic profile observed in obesity.

## Methods

### Study design and participants

This was a single-center, case–control study conducted at the Medical Research Institute, Alexandria University, Egypt, between January 2024 and March 2024. The study group comprised patients with obesity (BMI ≥ 35 kg/m²) who were scheduled to undergo laparoscopic sleeve gastrectomy (SG), while the control group included non-obese individuals (BMI < 30 kg/m²) undergoing diagnostic upper gastrointestinal endoscopy for non-inflammatory, unrelated indications.

Obesity was defined using a composite of anthropometric criteria: body mass index (BMI), waist circumference (WC), and waist-to-hip ratio (WHR). Participants were classified as having obesity if they had a BMI ≥ 35 kg/m², WC > 94 cm, and WHR > 1.0. This classification is consistent with the 2022 ASMBS/IFSO guidelines for surgical eligibility^18^. All individuals in the obesity group also exhibited at least one marker of metabolic dysregulation, such as elevated fasting glucose or HOMA-IR ^19^. Control participants had a BMI of < 30 kg/m², WC ≤ 94 cm, WHR ≤ 0.8, and normal fasting glucose and insulin sensitivity, indicating the absence of metabolic dysfunction^20^.

Exclusion criteria for both groups included known diabetes mellitus, hypertension, cardiovascular disease, hepatic or renal impairment, active malignancy, prior bariatric or gastrointestinal surgery, or the use of medications known to affect metabolic or hormonal regulation (e.g., corticosteroids, thiazolidinediones, ACE inhibitors, ARBs, clonidine, or fenofibrate). All participants provided written informed consent before enrollment. The study was approved by the Ethics Committee of the Medical Research Institute, Alexandria University (Approval No. E/C. S/N.R12/2024) and conducted in accordance with the Declaration of Helsinki^21^.

### Surgical technique

All SG procedures were performed laparoscopically using a standardized five-port technique: three 12-mm ports (camera, right and left working ports) and two 5-mm ports, for liver retraction and assistance access, respectively. Pneumoperitoneum was established using optical trocars. The greater omentum was dissected off the greater curvature of the stomach using the EnSeal device (Ethicon Endo-Surgery, Cincinnati, OH, USA), followed by mobilization of the posterior gastric wall and excision of Belsey’s fat pad. A 40-Fr orogastric calibration tube was positioned. Gastric division was performed using the Echelon Flex Endopath 60-mm linear stapler (Ethicon Endo-Surgery) starting 3–5 cm from the pylorus up to the angle of His. Stapler reloads were selected based on tissue thickness (green, gold, blue). The entire staple line was invaginated using running seromuscular sutures with 3/0 V-Loc 180 absorbable barbed sutures (Covidien, Mansfield, MA, USA). Incidental hiatal hernias, if identified intraoperatively, were repaired with posterior cruroplasty before gastric division.

### Gastric tissue collection and gene expression analysis

In the surgical group, full-thickness gastric biopsies were collected intraoperatively from the resected fundus along the greater curvature immediately following sleeve gastrectomy. In the control group, gastric biopsies were obtained endoscopically from the anterior wall of the fundus using standard biopsy forceps during upper gastrointestinal endoscopy. To minimize site-related variability, all specimens were taken from anatomically comparable fundic regions using a standardized sampling protocol. Only control participants with macroscopically and histologically normal gastric mucosa were included.

Total RNA was extracted from all tissue samples using the RNeasy Mini Kit (Qiagen, Hilden, Germany), and RNA purity was verified using NanoDrop spectrophotometry, with acceptable A260/A280 ratios between 1.8 and 2.0. Complementary DNA (cDNA) was synthesized using the QuantiTect Reverse Transcription Kit (Qiagen, Cat. No. 205311). Quantitative real-time PCR (qRT-PCR) was performed on a Rotor-Gene Q system using the QuantiNova SYBR Green PCR Kit (Qiagen, USA). Pre-designed LNA-based primers were used for the target genes FTO (SBH0350496) and MC4R (SBH0618803), with GAPDH (SBH0555536) serving as the reference gene, which showed stable expression across all samples and was used for normalization^22^. All reactions were performed in duplicate. The amplification protocol included an initial denaturation at 95 °C for 10 min, followed by 40 cycles of denaturation at 95 °C for 5 s, annealing at 55 °C for 15 s, and extension at 60 °C for 15 s. Relative gene expression was calculated using the 2^-ΔΔCT method^23,24^.

Melting-curve analyses confirmed single, specific amplicons for both target and reference genes, ensuring the absence of nonspecific amplification or primer-dimer artifacts. Cycle threshold (Ct) values were obtained for all genes as part of the qRT-PCR workflow and used solely to compute relative expression via the 2^–ΔΔCT method, which normalizes target-gene expression to the reference gene. As is standard in comparative qRT-PCR analyses, raw Ct values were not independently analyzed or presented, since biological interpretation derives from normalized relative expression rather than absolute amplification cycles.

### Anthropometric and biochemical measurements

All participants underwent standardized anthropometric measurements, including weight, height, WC, and WHR. BMI was calculated as weight (kg) divided by height in meters squared (m²). All participants fasted overnight for 8–10 h before venous blood sampling to ensure stable baseline metabolic and hormonal levels. Fasting venous blood samples were obtained intraoperatively before tissue collection.

Serum was aliquoted: one portion was used for assessment of fasting blood glucose (FBG), insulin, total cholesterol, LDL, HDL, and triglycerides; another portion was used to measure serum levels of leptin, adiponectin, and ghrelin. Hormonal assays were performed using ELISA kits (Chongqing Biospes Co., Ltd, China; Cat. Nos. BZEK2006, BZEK2289, BZEK2282) according to the manufacturer’s protocols. All measurements were done in duplicate. HOMA-IR was calculated as:

$$HOMA - IR{\text{ }} = {\text{ }}\left[ {fasting{\text{ }}insulin{\text{ }}\left( {\mu IU/mL} \right){\text{ }} \times {\text{ }}fasting{\text{ }}glu\cos e{\text{ }}\left( {mg/dL} \right)} \right]{\text{ }}/{\text{ }}405$$

### Sample size calculation

Sample size was determined using G*Power software (v3.1.9.7), assuming a large effect size (Cohen’s d = 0.80), an alpha level of 0.05, and 80% statistical power for two-sided comparisons. This yielded a minimum required sample of 52 participants (26 per group). To increase statistical reliability and allow for confounder adjustment, the study included 100 participants (50 per group).

### Statistical analysis

Descriptive statistics were used to summarize participant characteristics. Continuous variables were expressed as mean ± standard error (SE), and categorical variables as counts and percentages. Between-group comparisons were performed using linear regression for continuous outcomes and weighted chi-square tests for categorical variables.

To minimize confounding by age and sex, inverse propensity score weighting (IPSW) was employed using the TWANG package (Toolkit for Weighting and Analysis of Nonequivalent Groups). A binomial gradient boosting machine (GBM) model was applied in an Average Treatment Effect on the Treated (ATT) framework. Balance was assessed using absolute standardized mean differences (aSMD), with a threshold of < 0.1 considered optimal. Optimal balance was achieved at 240 GBM trees.

For metabolic, hormonal, and gene expression outcomes, both unadjusted and IPSW-adjusted mean differences (MD and AMD) were calculated with 95% confidence intervals. Additional regression models were performed post-weighting to adjust for residual age and sex effects (double adjustment). Associations between FTO/MC4R expression and metabolic parameters were examined using weighted linear regression, controlling for obesity status. Because conventional OLS R² statistics are not directly interpretable under IPSW, we additionally computed survey-weighted pseudo-R² values for each weighted regression model to summarize overall fit, using the formula

$$\:{R}_{w}^{2}=1-\frac{\sum\:{w}_{i}({y}_{i}-{\widehat{y}}_{i}{)}^{2}}{\sum\:{w}_{i}({y}_{i}-{\stackrel{\prime }{y}}_{w}{)}^{2}}$$

These values are reported in Table 3 for transparency. All statistical analyses were conducted using R software (v4.2.1), and significance was set at *p* < 0.05.

**Table 3:** Associations of FTO and MC4R gene expression with metabolic and hormonal outcomes after adjusting for case status using inverse propensity score Weighting.

Outcome	Covariate	FTO			MC4R		
		MD (95% CI)	p	R2	MD (95% CI)	p	R2
BMI (Kg/m^2^)	Expr	0.04 (-0.61, 0.69)	0.903	0.73	-0.46 (-2.81, 1.89)	0.701	0.73
	Case	17.48 (13.83, 21.14)	< 0.001*		17.25 (14.63, 19.87)	< 0.001*	
Total lipid (mg/dL)	Expr	3.12 (-4.72, 10.96)	0.432	0.01	29.18 (-43.38, 101.74)	0.427	0.01
	Case	-24.19 (-79.40, 31.01)	0.387		17.55 (-55.98, 91.08)	0.637	
Triglycerides (mg/dL)	Expr	-0.68 (-4.77, 3.42)	0.744	0.00	-9.52 (-30.21, 11.17)	0.363	0.01
	Case	-1.24 (-26.46, 23.98)	0.922		-13.23 (-37.86, 11.40)	0.289	
Cholesterol (mg/dL)	Expr	2.07 (-0.36, 4.49)	0.095	0.02	20.52 (-9.32, 50.36)	0.175	0.03
	Case	-7.26 (-28.02, 13.51)	0.490		21.48 (-7.55, 50.52)	0.145	
LDL (mg/dL)	Expr	1.94 (-0.23, 4.10)	0.079	0.02	20.90 (-5.12, 46.92)	0.114	0.04
	Case	-9.61 (-27.54, 8.32)	0.290		18.87 (-5.44, 43.17)	0.127	
HDL (mg/dL)	Expr	0.23 (-0.47, 0.93)	0.518	0.06	0.34 (-6.69, 7.37)	0.924	0.06
	Case	3.87 (-2.19, 9.93)	0.207		5.27 (-1.58, 12.12)	0.130	
Cholesterol/HDL	Expr	0.03 (-0.03, 0.09)	0.348	0.05	-0.21 (-1.00, 0.58)	0.592	0.05
	Case	0.39 (-0.29, 1.07)	0.261		0.33 (-0.54, 1.20)	0.451	
LDL/HDL	Expr	0.02 (-0.02, 0.07)	0.296	0.05	0.37 (-0.32, 1.05)	0.293	0.06
	Case	-0.45 (-0.90, 0.01)	0.057		0.00 (-0.61, 0.61)	0.993	
Insulin (micro IU/mL)	Expr	-0.21 (-0.91, 0.50)	0.564	0.02	3.82 (-0.48, 8.12)	0.081	0.05
	Case	-0.95 (-6.52, 4.63)	0.737		1.59 (-2.61, 5.80)	0.454	
HOMA-IR	Expr	-0.06 (-0.23, 0.11)	0.498	0.01	1.08 (0.01, 2.15)	0.048*	0.05
	Case	-0.04 (-1.37, 1.30)	0.954		0.68 (-0.39, 1.74)	0.209	
FBG (mg/dL)	Expr	-0.25 (-1.24, 0.75)	0.626	0.05	8.74 (-0.87, 18.36)	0.074	0.08
	Case	8.32 (-0.09, 16.73)	0.052		15.22 (4.93, 25.51)	0.004*	
Leptin (ng/mL)	Expr	-0.08 (-0.27, 0.10)	0.377	0.03	-0.04 (-0.66, 0.59)	0.911	0.01
	Case	0.69 (-0.58, 1.97)	0.285		0.26 (-0.38, 0.90)	0.421	
Adiponectin (mcg/mL)	Expr	-4.73 (-25.97, 16.52)	0.660	0.28	-49.38 (-148.94, 50.19)	0.327	0.28
	Case	219.35 (111.14, 327.55)	< 0.001*		151.33 (43.88, 258.79)	0.006*	
Ghrelin (pg/mL)	Expr	-4.14 (-13.77, 5.49)	0.396	0.03	27.93 (-18.84, 74.69)	0.239	0.03
	Case	-9.15 (-89.77, 71.46)	0.822		-3.11 (-46.76, 40.53)	0.888	

MD = Mean Difference; CI = Confidence Interval; Expr = Estimated mean difference in each outcome per unit increase in gene expression (FTO or MC4R), adjusted for case status; Case = Estimated mean difference between obese (cases) and non-obese (controls), adjusted for gene expression. Results are derived from inverse propensity-score-weighted (IPSW) linear regression models including both predictors simultaneously. Statistically significant *p*-values (*p* < 0.05) are indicated with an asterisk.

## Results

### Participant characteristics

Before weighting, patients with obesity were significantly younger than controls (31.4 ± 1.5 vs. 39.2 ± 2.1 years; *p* = 0.003), with an absolute standardized mean difference (aSMD) of 0.75 (Table 1). After inverse propensity score weighting (IPSW), age imbalance improved (*p* = 0.284; aSMD = 0.28), though the ideal threshold of < 0.1 was not reached. Sex distribution was similar between groups (*p* = 0.469), and weighting further minimized this difference (aSMD = 0.01). As expected, body weight and BMI were significantly higher in the obesity group (118.3 ± 3.2 vs. 68.9 ± 1.5 kg; BMI 43.1 ± 0.9 vs. 25.9 ± 0.4 kg/m²; both *p* < 0.001), and these differences were preserved post-weighting (Table 1).

**Table 1:** Demographic data of the participants.

Variable	Unadjusted analysis				IPSW adjusted analysis			
	Cases *n* = 50	Controls *n* = 50	*p*	aSMD	Cases *n* = 50	Controls *n* = 35	*p*	aSMD
Age	31.4 ± 1.5	39.2 ± 2.1	0.003*	0.75	31.4 ± 1.5	34.3 ± 2.3	0.284	0.28
Sex								
Male	9 (18)	13 (26)	0.469	0.21	9 (18)	6 (18.2)	0.978	0.01
Female	41 (82)	37 (74)			41 (82)	29 (81.8)		
Anthropometrics								
Height (cm)	165.4 ± 1.3	162.8 ± 0.9	0.106	0.33	165.4 ± 1.3	162.7 ± 1.3	0.139	0.33
Weight (Kg)	118.3 ± 3.2	68.9 ± 1.5	< 0.001*	2.83	118.3 ± 3.2	67.6 ± 2.3	< 0.001*	2.84
BMI (Kg/m^2^)	43.1 ± 0.9	25.9 ± 0.4	< 0.001*	3.39	43.1 ± 0.9	25.4 ± 0.6	< 0.001*	3.45

Cell values represent Mean ± standard errors. aSMD: absolute standardized mean difference; IPSW: Inverse Propensity Score Weight; *: Statistically significant.

### Metabolic profile

Patients with obesity had significantly higher fasting blood glucose (FBG; MD = 10.88 mg/dL; *p* = 0.003) and high-density lipoprotein (HDL) levels (MD = 7.28 mg/dL; *p* < 0.001), and a lower LDL/HDL ratio (MD = − 0.56; *p* = 0.001) (Table 2). After IPSW, FBG (AMD = 7.48; *p* = 0.018) and HDL (AMD = 4.54; *p* = 0.042) remained significantly different, while the LDL/HDL ratio difference became non-significant (*p* = 0.061). There were no significant group differences in triglycerides, insulin, or HOMA-IR before or after weighting (Table 2).

**Table 2:** Differences between obese cases and non-obese controls in terms of their metabolic profile, appetite, and metabolic hormones, and genetic expression of FTO and MC4R.

Variable	Unadjusted analysis			IPSW adjusted analysis		
	Cases *n* = 50	Controls *n* = 50	MD (95% CI)	Cases *n* = 50	Controls *n* = 35	AMD (95% CI)
Metabolic profile						
Total lipid (mg/dL)	421.4 ± 13.2	434.3 ± 16.0	-12.88 (-54.01, 28.24)	421.4 ± 13.1	434.3 ± 18.1	-12.84 (-57.18, 31.50)
Triglycerides (mg/dL)	105.8 ± 5.4	111.0 ± 6.8	-5.20 (-22.44, 12.04)	105.8 ± 5.4	108.1 ± 7.1	-2.34 (-20.06, 15.38)
Cholesterol (mg/dL)	174.2 ± 4.6	172.9 ± 7.1	1.26 (-15.47, 17.99)	174.2 ± 4.6	174.2 ± 7.9	-0.04 (-18.18, 18.10)
LDL (mg/dL)	104.6 ± 3.6	108.8 ± 6.1	-4.20 (-18.21, 9.81)	104.6 ± 3.6	107.6 ± 7.1	-2.98 (-18.85, 12.88)
HDL (mg/dL)	48.8 ± 1.4	41.5 ± 1.4	**7.28 (3.41**, 11.14)*	48.8 ± 1.4	44.3 ± 1.7	**4.54 (0.17**, 8.91)*
Cholesterol/HDL	3.3 ± 0.1	2.9 ± 0.2	0.40 (-0.10, 0.91)	3.3 ± 0.1	2.7 ± 0.3	**0.61 (0.06**, 1.16)*
LDL/HDL	2.2 ± 0.1	2.8 ± 0.1	-**0.56 (-0.89**, -0.24)*	2.2 ± 0.1	2.6 ± 0.2	-0.38 (-0.78, 0.02)
Insulin (micro IU/mL)	8.7 ± 1.2	9.6 ± 0.6	-0.94 (-3.54, 1.66)	8.7 ± 1.2	10.5 ± 1.0	-1.83 (-4.88, 1.22)
HOMA-IR	2.1 ± 0.3	2.1 ± 0.2	-0.02 (-0.67, 0.62)	2.1 ± 0.3	2.3 ± 0.2	-0.29 (-1.02, 0.45)
FBG (mg/dL)	94.7 ± 1.9	83.8 ± 3.0	**10.88 (3.89**, 17.87)*	94.7 ± 1.9	87.2 ± 2.5	**7.48 (1.29**, 13.68)*
Appetite and Metabolic Hormones						
Leptin (ng/mL)	3.4 ± 0.3	3.1 ± 0.1	0.22 (-0.36, 0.80)	3.4 ± 0.3	3.1 ± 0.1	0.25 (-0.33, 0.82)
Adiponectin (mcg/mL)	430.5 ± 28.1	228.1 ± 10.5	202.34 **(142.76**, 261.92)*	430.5 ± 28.0	235.3 ± 12.2	195.18 **(134.63**, 255.74)*
ghrelin (pg/mL)	205.5 ± 17.0	221.4 ± 10.1	-15.94 (-55.23, 23.35)	205.5 ± 17.0	234.3 ± 12.0	-28.80 (-70.08, 12.49)
Genetic expression						
FTO (fold change)	5.8 ± 0.4	0.9 ± 0.1	**4.82 (3.97**, 5.67)*	5.8 ± 0.4	1.0 ± 0.1	**4.76 (3.92**, 5.61)*
MC4R (fold change)	0.1 ± 0.0	1.0 ± 0.1	**-0.83 (-0.96**, -0.70)*	0.1 ± 0.0	1.0 ± 0.1	**-0.91 (-1.08**, -0.74)*

IPSW: Inverse Propensity Score Weight; MD: Mean Difference; AMD: Adjusted Mean Difference; CI: Confidence Interval; *: Statistically significant.

### Appetite-related hormones

**Fig. 1:**
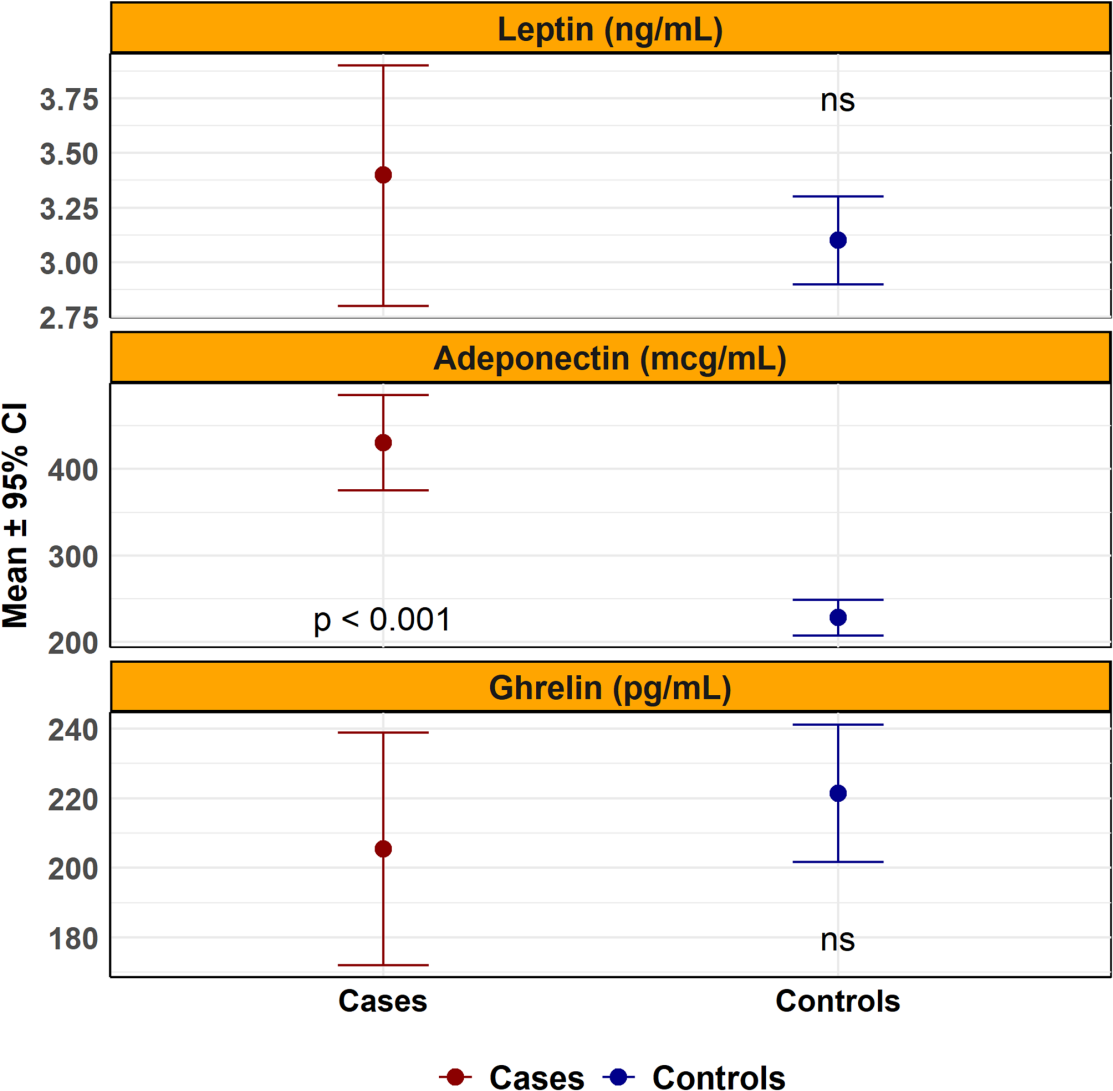
Serum concentrations of leptin, adiponectin, and ghrelin in patients with and without obesity. Mean serum levels of leptin (ng/mL), adiponectin (mcg/mL), and ghrelin (pg/mL) are shown with 95% confidence intervals. Adiponectin was significantly higher in patients with obesity (p < 0.001), while no significant differences were observed for leptin or ghrelin. Hormone levels were measured using ELISA. Group comparisons were performed using weighted linear regression. Red and blue dots represent group means for patients with obesity and controls, respectively. Ns: not statistically significant.

Adiponectin levels were significantly higher in the obesity group both before (MD = 202.34 µg/mL; *p* < 0.001) and after weighting (AMD = 195.18 µg/mL; *p* < 0.001) (Fig. 1). There were no significant between-group differences in leptin or ghrelin levels in either unadjusted or adjusted models (Table 2).

### Gastric gene expression

**Fig. 2:**
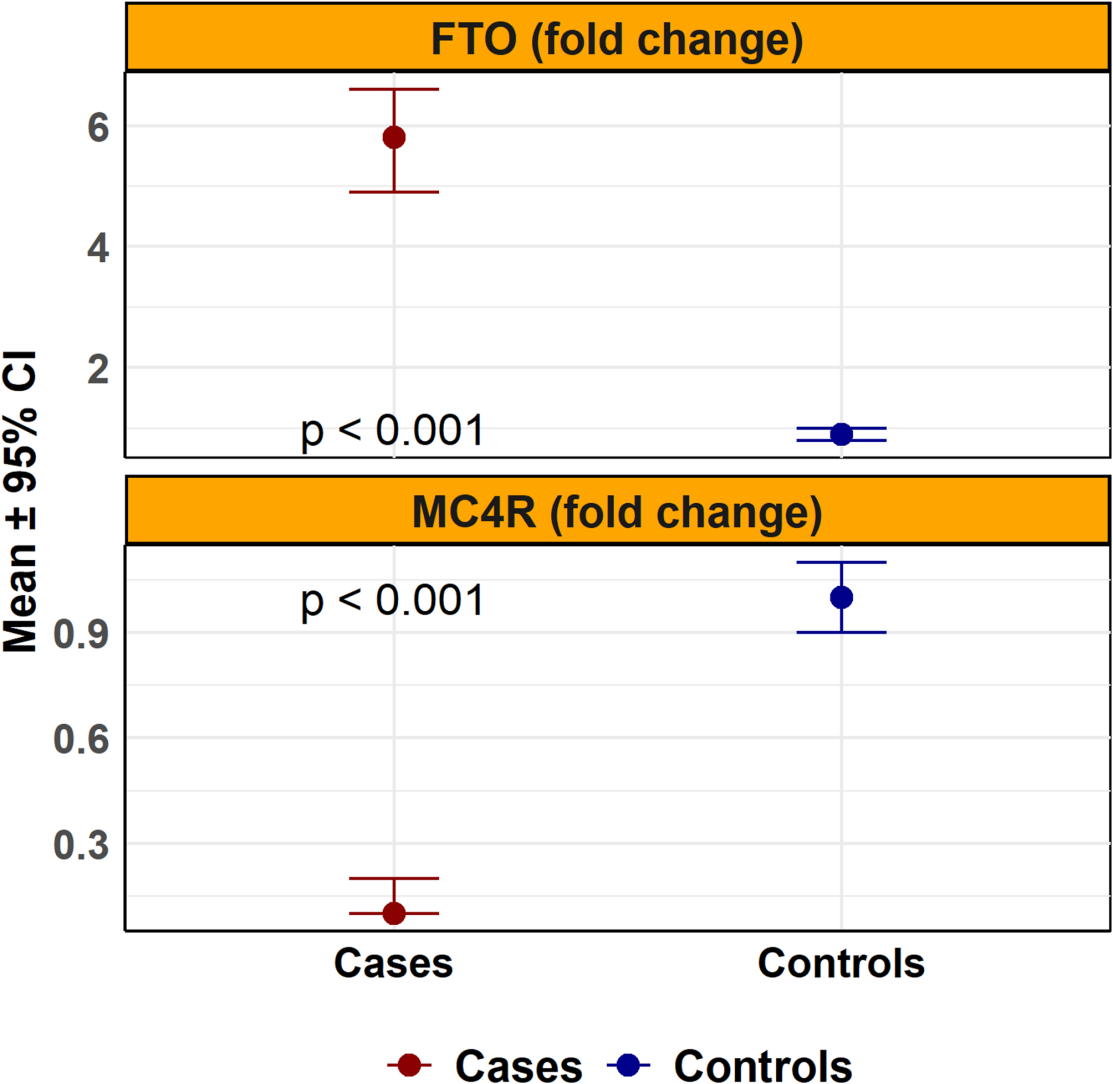
Relative gastric expression of FTO and MC4R genes in patients with and without obesity.Mean fold-change (2^−ΔΔCT) in gastric expression of FTO and MC4R genes, normalized to GAPDH, is presented with 95% confidence intervals. FTO was significantly upregulated and MC4R was significantly downregulated in patients with obesity compared to controls (both p < 0.001). Expression was measured by quantitative real-time PCR. Group comparisons were conducted using weighted linear regression. Red and blue dots indicate mean values for the obesity and control groups, respectively.

FTO expression was significantly upregulated in the obesity group (fold-change: 5.8 vs. 1.0; *p* < 0.001), while MC4R expression was markedly downregulated (fold-change: 0.1 vs. 1.0; *p* < 0.001) (Fig. 2). These differences remained highly significant after weighting (AMD for FTO = 4.76; *p* < 0.001 and AMD for MC4R = − 0.91; *p* < 0.001) (Table 2).

### Associations between gene expression and metabolic parameters

Regression models adjusted for case status (Table 3) showed that higher gastric MC4R expression was positively associated with HOMA-IR (β = 1.08, *p* = 0.048). This finding reflects an individual-level association across the entire cohort, as the model included both gene expression and obesity status simultaneously. By contrast, the between-group comparison of mean HOMA-IR (Table 2) did not show a significant difference between obese and non-obese participants. These results are therefore not contradictory: HOMA-IR in obesity is determined by multiple metabolic influences (e.g., inflammatory and adipokine-mediated mechanisms), so the group average may remain unchanged even when a positive adjusted relationship between MC4R expression and HOMA-IR is detectable at the individual level.

### Hormone–metabolism associations

Associations between serum hormones and metabolic outcomes across all participants revealed that Adiponectin was significantly associated with higher BMI (β = 0.03; *p* < 0.001) and increased HDL cholesterol (*p* = 0.026) in multivariable-adjusted models. Leptin and ghrelin showed no significant associations with insulin resistance, glucose, or lipid profile (Table 4).

**Table 4:** Unadjusted and adjusted associations between serum levels of Leptin, Adiponectin, and Ghrelin and various metabolic Parameters.

Outcomes	Leptin (ng/mL)		Adiponectin (mcg/mL)		Ghrelin (pg/mL)	
	Unadjusted	Adjusted	Unadjusted	Adjusted	Unadjusted	Adjusted
	β (95% CI)	β (95% CI)	β (95% CI)	β (95% CI)	β (95% CI)	β (95% CI)
BMI (Kg/m^2^)	-0.20 (-1.58, 1.17)	-0.94 (-2.93, 1.05)	**0.03 (0.02**, 0.04)*	**0.03 (0.01**, 0.04)*	-0.01 (-0.03, 0.01)	-0.00 (-0.03, 0.03)
Total lipid (mg/dL)	0.86 (-13.38, 15.10)	-3.68 (-26.07, 18.71)	-0.04 (-0.16, 0.07)	0.01 (-0.11, 0.14)	-0.01 (-0.22, 0.20)	0.02 (-0.29, 0.33)
Triglycerides (mg/dL)	-3.03 (-8.97, 2.91)	-1.91 (-11.36, 7.53)	-0.04 (-0.09, 0.01)	-0.02 (-0.08, 0.03)	-0.03 (-0.12, 0.05)	-0.02 (-0.15, 0.11)
Cholesterol (mg/dL)	2.64 (-3.11, 8.40)	0.41 (-8.66, 9.47)	0.01 (-0.04, 0.06)	0.03 (-0.02, 0.08)	0.01 (-0.08, 0.09)	0.00 (-0.12, 0.13)
LDL (mg/dL)	2.84 (-1.97, 7.66)	2.06 (-5.52, 9.65)	-0.00 (-0.04, 0.04)	0.01 (-0.04, 0.05)	0.01 (-0.06, 0.09)	-0.01 (-0.11, 0.10)
HDL (mg/dL)	0.43 (-0.99, 1.86)	-1.53 (-3.67, 0.62)	**0.02 (0.01**, 0.03)*	**0.03 (0.01**, 0.04)*	0.00 (-0.02, 0.02)	0.02 (-0.01, 0.05)
Cholesterol/HDL	-0.01 (-0.19, 0.16)	0.21 (-0.08, 0.49)	-0.00 (-0.00, 0.00)	-0.00 (-0.00, 0.00)	-0.00 (-0.00, 0.00)	-0.00 (-0.01, 0.00)
LDL/HDL	0.05 (-0.07, 0.17)	0.14 (-0.04, 0.32)	-0.00 (-0.00, 0.00)	-0.00 (-0.00, 0.00)	-0.00 (-0.00, 0.00)	-0.00 (-0.00, 0.00)
Insulin (micro IU/mL)	-0.48 (-1.38, 0.41)	0.09 (-1.32, 1.50)	0.00 (-0.00, 0.01)	0.00 (-0.00, 0.01)	-0.01 (-0.02, 0.00)	-0.01 (-0.03, 0.01)
HOMA-IR	-0.09 (-0.31, 0.14)	0.03 (-0.32, 0.38)	0.00 (-0.00, 0.00)	0.00 (-0.00, 0.00)	-0.00 (-0.01, 0.00)	-0.00 (-0.01, 0.00)
FBG (mg/dL)	0.99 (-1.53, 3.52)	-0.58 (-4.70, 3.55)	0.02 (-0.00, 0.04)	0.02 (-0.00, 0.05)	0.01 (-0.03, 0.05)	0.01 (-0.05, 0.07)

* Statistically significant at *p* < 0.05. β coefficients represent the estimated change in each metabolic parameter per unit increase in serum hormone concentration (leptin, adiponectin, or ghrelin). Unadjusted models include each hormone separately without covariates. Adjusted models are controlled for age, sex, and the other two hormones simultaneously. BMI = Body Mass Index; LDL = Low-Density Lipoprotein; HDL = High-Density Lipoprotein; HOMA-IR = Homeostasis Model Assessment of Insulin Resistance; FBG = Fasting Blood Glucose.

Within the obesity subgroup (Table 5), higher leptin levels were paradoxically associated with lower BMI (β = −2.33; *p* = 0.016). No other hormone–metabolism associations were statistically significant among patients with obesity after adjustment.

**Table 5:** Unadjusted and adjusted associations between serum levels of Leptin, Adiponectin, and Ghrelin and various metabolic parameters among obese subjects.

Outcomes	Leptin (ng/mL)		Adiponectin (mcg/mL)		Ghrelin (pg/mL)	
	Unadjusted	Adjusted	Unadjusted	Adjusted	Unadjusted	Adjusted
	β (95% CI)	β (95% CI)	β (95% CI)	β (95% CI)	β (95% CI)	β (95% CI)
BMI (Kg/m^2^)	-0.74 (-1.69, 0.20)	**-2.33 (-4.25**, -0.42)*	-0.00 (-0.01, 0.01)	0.00 (-0.01, 0.01)	-0.00 (-0.02, 0.01)	0.03 (-0.00, 0.06)
Total lipid (mg/dL)	-2.96 (-16.99, 11.07)	0.30 (-25.28, 25.88)	-0.07 (-0.20, 0.07)	-0.03 (-0.17, 0.11)	-0.06 (-0.29, 0.16)	-0.06 (-0.45, 0.33)
Triglycerides (mg/dL)	-1.91 (-7.64, 3.81)	-0.70 (-11.75, 10.35)	-0.04 (-0.09, 0.02)	-0.03 (-0.09, 0.03)	-0.03 (-0.12, 0.07)	-0.02 (-0.18, 0.15)
Cholesterol (mg/dL)	0.26 (-4.65, 5.18)	2.18 (-6.86, 11.23)	-0.01 (-0.06, 0.04)	0.00 (-0.05, 0.05)	-0.01 (-0.09, 0.06)	-0.04 (-0.17, 0.10)
LDL (mg/dL)	0.69 (-3.18, 4.55)	2.08 (-5.02, 9.19)	-0.01 (-0.05, 0.03)	-0.01 (-0.04, 0.03)	-0.00 (-0.06, 0.06)	-0.02 (-0.13, 0.09)
HDL (mg/dL)	-0.01 (-1.47, 1.45)	0.24 (-2.70, 3.17)	0.01 (-0.00, 0.03)	0.01 (-0.00, 0.03)	-0.01 (-0.03, 0.01)	-0.01 (-0.06, 0.03)
Cholesterol/HDL	0.01 (-0.11, 0.13)	0.05 (-0.19, 0.28)	-0.00 (-0.00, 0.00)	-0.00 (-0.00, 0.00)	0.00 (-0.00, 0.00)	0.00 (-0.00, 0.00)
LDL/HDL	0.02 (-0.07, 0.10)	0.06 (-0.10, 0.23)	-0.00 (-0.00, 0.00)	-0.00 (-0.00, 0.00)	0.00 (-0.00, 0.00)	-0.00 (-0.00, 0.00)
Insulin (micro IU/mL)	-0.52 (-1.77, 0.73)	0.34 (-1.94, 2.63)	0.00 (-0.01, 0.02)	0.00 (-0.01, 0.01)	-0.01 (-0.03, 0.01)	-0.02 (-0.06, 0.01)
HOMA-IR	-0.11 (-0.41, 0.19)	0.16 (-0.39, 0.71)	0.00 (-0.00, 0.00)	-0.00 (-0.00, 0.00)	-0.00 (-0.01, 0.00)	-0.01 (-0.01, 0.00)
FBG (mg/dL)	0.22 (-1.80, 2.24)	2.50 (-1.59, 6.58)	-0.02 (-0.03, 0.00)	-0.02 (-0.04, 0.00)	-0.00 (-0.03, 0.03)	-0.03 (-0.09, 0.03)

* Statistically significant at *p* < 0.05. β coefficients represent the estimated change in each metabolic parameter per unit increase in serum hormone concentration (leptin, adiponectin, or ghrelin). Unadjusted models include each hormone separately without covariates. Adjusted models are controlled for age, sex, and the other two hormones simultaneously. BMI = Body Mass Index; LDL = Low-Density Lipoprotein; HDL = High-Density Lipoprotein; HOMA-IR = Homeostasis Model Assessment of Insulin Resistance; FBG = Fasting Blood Glucose.

## Discussion

This study examined the relationship between appetite-regulating hormones and the expression of FTO and MC4R genes in the gastric tissue of patients with and without obesity. Our findings emphasize the importance of local gastric gene expression in obesity and suggest that peripheral molecular changes may lead to systemic metabolic dysregulation. To our knowledge, this is the first study to concurrently assess gastric FTO and MC4R gene expression alongside circulating adipokines in humans, offering new insights into these genes’ roles in obesity.

Key hormones such as leptin, adiponectin, and ghrelin have been implicated in the pathogenesis of obesity, irrespective of their site of secretion or mechanisms of action^6,9–11^. Studies have thoroughly investigated their roles concerning obesity-related complications, including cardiovascular disease, metabolic disorders, and malignancies^6–8^.

Genetically, the Fat Mass and Obesity-Associated (FTO) gene and the Melanocortin-4 Receptor (MC4R) gene are recognized as contributors to polygenic and monogenic obesity, respectively^13,14^. Numerous genetic studies have revealed associations between specific polymorphisms and an elevated risk of obesity, including the rs9939609 SNP of FTO, which correlates with obesity and heightened adiponectin levels^25^. Additionally, polymorphisms rs17782313 and rs12970134 of the MC4R gene have also shown relevance^17^.

Epigenetic investigations into FTO’s association with obesity have produced conflicting results^26,27^. Methylation studies of the MC4R gene indicate a connection to metabolic syndrome, yet show no direct link to obesity^28–30^. Most genetic expression analyses of these genes have been conducted using murine models, cell lines, and umbilical cord blood samples^31–33^.

We observed significant upregulation of FTO and downregulation of MC4R in the gastric tissue of patients with obesity, correlating with altered adiponectin levels and insulin resistance. These observations are consistent with earlier research showing that single-nucleotide polymorphisms (SNPs) in the FTO gene, particularly rs9939609, are strongly associated with increased risk of obesity, unfavorable lipid profiles, and higher adiponectin concentrations in obese individuals^25,34–37^. Other SNPs such as rs1421085, rs9939973, rs8050136, rs1781749, and rs3751812 have shown variable associations with adiposity risk^35,36^. Expression studies in knockout mouse models have demonstrated that deletion of FTO protects against diet-induced obesity^31^. Moreover, FTO expression has been confirmed in embryonic tissue and across multiple organs, including the central nervous system, myocardium, cerebellum, kidney, and salivary glands^32^, suggesting its systemic regulatory role^31–33^.

The correlation of adiponectin with BMI further implicates FTO in obesity-related complications^38^. Our results aligned with a previous study demonstrating a positive correlation between FTO and PLAG1 gene expression in the peripheral blood of children with BMI values, where serum concentrations of adipokines and leptin were positively correlated, while apelin and the leptin receptor demonstrated negative correlations^27^. This suggests the involvement of FTO in the pathophysiology of obesity-related adiposity^27^.

Additionally, FTO gene expression in peripheral blood has been linked to childhood obesity, showing positive correlations with fasting free fatty acid and insulin levels during glucose tolerance tests. Likewise, FTO methylation exhibited a positive correlation with triglyceride levels and fasting insulin^39^. These findings are consistent with our results.

The relationship between FTO activity and adiponectin concentration remains controversial across studies. Some investigations have reported lower adiponectin levels in carriers of risk alleles, whereas others, particularly those examining gestational or pediatric obesity, describe elevated or preserved adiponectin concentrations despite increased FTO expression^40,41^. Our findings contribute to this evolving evidence by suggesting that enhanced gastric FTO expression may coexist with elevated circulating adiponectin, consistent with the concept of the adiponectin paradox and the metabolically healthy obesity phenotype. Recent work further supports that central and peripheral melanocortin signaling (via MC4R) modulates insulin sensitivity and adipokine balance, lending mechanistic plausibility to our observations^42,43^.

Studies have demonstrated that the expression of the FTO protein is modulated by both leptin and IL-6. Furthermore, in vitro studies have shown that overexpression of FTO can influence the phosphorylation status of leptin-stimulated STAT3 at Y705 and S727. This alteration contributes to the dysregulation of glucose-6-phosphatase (G6P) expression^44^. Additionally, a study on piglets enhances our understanding of the role of the FTO gene in the pathogenesis of obesity-related adiposity. It demonstrates that FTO expression is upregulated in response to leptin, leading to alterations in lipid metabolism and changes in energy expenditure^45^.

A recent study identified that the allelic frequencies of the fat mass and obesity-associated (FTO) polymorphism FTO rs9939609 (A) and the leptin receptor polymorphism LEPR rs1137101 (223R) are correlated with an elevated risk of obesity among the Egyptian population^45,46^. There is a link between FTO SNP rs9939609, adiponectin levels, and physical activity, suggesting that FTO variants relate to central obesity, potentially influenced by lifestyle^37^. Different FTO genotypes also affect FTO expression levels in adipose and skeletal muscle tissues^47^.

MC4R, traditionally seen as a central hypothalamic receptor, is also expressed in peripheral tissues like the stomach, pancreas, and adipose tissue, affecting energy expenditure and autonomic signaling. In animal models, peripheral MC4R expression has been identified in enteroendocrine L cells of the gut, where it regulates secretion of peptide YY and GLP-1, thereby linking melanocortin signaling to gut–brain communication and glucose homeostasis^48^. Similarly, Wei et al. ^49^ confirmed MC4R expression in both central and peripheral systems, reinforcing its dual role in metabolic regulation. Our detection of gastric MC4R transcripts in human tissue therefore aligns with this expanding evidence, supporting the concept that peripheral melanocortin signaling contributes to metabolic regulation beyond central pathways. In the present study, the expression of the MC4R gene in gastric tissue demonstrated a significant correlation with HOMA-IR, fasting blood glucose levels, and adiponectin concentrations. These findings support prior research suggesting a link between MC4R expression and its potential role in the pathophysiology of obesity-related type 2 diabetes mellitus^50^. The reduced expression of MC4R in obese individuals compared with controls may be related to its predominant localization in the dorsal motor nucleus of the vagus, which houses the preganglionic parasympathetic vagal efferent neurons pivotal to gastrointestinal regulation. This study represents the first investigation into gastric MC4R gene expression in human tissue^48^.

Although mean HOMA-IR values did not differ significantly between obese and control participants, the individual-level positive association between gastric MC4R expression and HOMA-IR suggests that this receptor’s expression reflects intra-group variability in insulin sensitivity rather than group-level differences. HOMA-IR in obesity is influenced by multiple mechanisms, including inflammatory, adipokine-mediated, and neuroendocrine pathways, so the group average may remain unchanged even when a positive adjusted correlation is detectable at the individual level. This interpretation underscores the multifactorial interplay between peripheral melanocortin signaling and systemic insulin regulation.

The SNP analysis of the MC4R gene indicated that the rs17782313 polymorphism is correlated with obesity, particularly in conjunction with the LEP GG genotype. These associations appear to be exacerbated by advancing age and mitigated by levels of physical activity^51^. Studies conducted on MC4R knockout mice revealed that the absence of MC4R correlates with reduced adiponectin levels, contributing to insulin resistance and heightened inflammation in adipose tissue^52^.

The investigation into MC4R polymorphisms highlighted that carriers of the MC4R variant rs17782313 exhibited a significantly elevated BMI (P-value = 0.046) and increased plasma ghrelin levels (P-value = 0.05). Specifically, individuals with TC or CC genotypes had markedly higher ghrelin concentrations compared to TT genotype carriers, with levels of 586.8 ± 218 pg/ml and 508 ± 207 pg/ml, respectively (P-value = 0.0197). This association of the MC4R variant with enhanced ghrelin levels suggests an impairment in MC4R functionality, which is critical for appetite regulation. Notably, ghrelin’s role involves inhibiting the activity of POMC neurons. Consequently, the non-significant difference in ghrelin levels observed between cases and controls (P-value = 0.169) may account for the reduced expression of MC4R in our cohort of obese patients. Furthermore, it’s important to note that ghrelin quantification should be addressed closely specially when using bead-based multiplexing technology, which may influence the sensitivity of the assay results^53^. The aforementioned study also reported no association between the MC4R rs17782313 variant and levels of either leptin or adiponectin.

In our analysis, after controlling for age, sex, and the two other hormones, we found that elevated leptin levels were significantly correlated with reduced BMI (β = -2.33, 95% CI: -4.25 to -0.42) specifically in the cohort of obese participants. Notably, no other significant relationships were detected for leptin, adiponectin, or ghrelin in relation to lipid profiles, insulin resistance indicators, or fasting blood glucose levels, whether in unadjusted or adjusted models. This outcome stands in contrast to existing literature that reports a robust positive correlation between serum leptin concentrations and body fat percentage. This discrepancy may be attributable to our relatively limited sample size, and it is important to note that neither the cases nor the controls exhibited insulin resistance or had a diabetes diagnosis^54^. In addition, all cases and controls were fasting and samples were collected preoperatively, as findings indicate that leptin levels decrease during fasting^55^and subsequently rise in response to feeding and surgical interventions^56^.

Persistent hyperinsulinemia has been shown to elevate leptin levels in humans, suggesting that leptin secretion is influenced by glucose metabolic pathways. A high-fat diet may disrupt glucose metabolism, potentially leading to increased adiposity and obesity. Leptin appears to modulate satiety through hypothalamic mechanisms, and there exists a negative feedback loop between the brain and adipose tissue that regulates this process^54^. The hypothalamic mechanism of leptin action involves specific signaling pathways mediated by the interaction with melanocortin receptors^52^. Our findings enhance the understanding of the primary mechanism by which leptin regulates body weight, specifically through the leptin-melanocortin signaling pathway.

In the present study Adiponectin levels were markedly higher in cases than in controls in the unadjusted analysis and in the IPSW-adjusted analysis with a MD = 202.34, 95% CI: 142.76 to 261.92, *p* < 0.001 and an AMD = 195.18, 95% CI: 134.63 to 255.74, *p* < 0.001, respectively. In unadjusted analyses, higher adiponectin levels were significantly associated with increased BMI and HDL cholesterol levels in the whole both groups.

Adiponectin plays a critical role in the pathophysiology of central obesity and its related comorbidities, particularly type 2 diabetes and cardiovascular diseases. This adipokine exerts its effects through specific adiponectin receptors, which modulate various metabolic pathways. Numerous studies have demonstrated that the administration of adiponectin, whether in human subjects or rodent models, elicits insulin-sensitizing, anti-atherogenic, and anti-inflammatory responses, alongside reductions in body weight^57–59^. Consequently, adiponectin has emerged as a promising target for replacement therapy aimed at managing obesity and its associated complications^57–59^.

The elevation of adiponectin observed among individuals with obesity in our cohort, both unadjusted and after weighting, may reflect a compensatory, anti-inflammatory, and insulin-sensitizing response characteristic of metabolically intact or metabolically healthy obesity phenotypes^57–59^. Interestingly, although obesity is typically associated with reduced HDL concentrations, our obese cohort exhibited modestly higher HDL-C levels. This paradox aligns with the metabolically healthy obesity (MHO) phenotype, in which individuals retain a favorable lipid profile despite excess adiposity^60,61^. Elevated adiponectin, as observed in this study, has been shown to correlate positively with HDL-C and to promote anti-atherogenic lipoprotein remodeling^62^. Together, these findings suggest that our cohort may represent an MHO-predominant population, characterized by preserved adiponectin activity and relatively protective lipid metabolism.

Adiponectin shows well-established correlations with HDL cholesterol and favorable lipoprotein particle distribution^62–64^, and genetic polymorphisms in the adiponectin gene have been linked to atherosclerosis and coronary artery disease risk^65^. Mechanistically, obesity-induced alterations in the adipose-tissue microenvironment and chronic low-grade inflammation can modulate adiponectin secretion and receptor dynamics^66^, while impaired receptor signaling or cleavage of T-cadherin reduces tissue sequestration and increases circulating levels, a process termed adiponectin resistance^67^. These adaptive changes are consistent with the adiponectin paradox, whereby some individuals with obesity, particularly those without overt diabetes, maintain or even enhance adiponectin secretion as a compensatory mechanism to preserve metabolic homeostasis^60,68–70^. Collectively, the evidence from Woodward et al. ^68^, Blüher^60^, Aguilar-Salinas et al. ^69^, Kalkman^67^, Fuster et al. ^68^, Han et al. ^71^, and Ahl et al. ^70^ supports the interpretation that elevated adiponectin in obesity represents a physiological adaptation rather than a contradiction of prior literature.

In addition, another study found that adiponectin was positively associated with LDL-P size, HDL-P size, and HDL-C and negatively with triglycerides, small LDL-P, large very LDL-P, and small HDL-P in obese patients^62^, aligning with our results. The correlation identified between adiponectin levels and HDL cholesterol specifically in pregnant women^72^, suggests that adiponectin may serve as a potential biomarker for gestational diabetes. Additionally, *Izadi et al.* conducted a systematic review of multiple studies, revealing that serum adiponectin levels are negatively correlated with triglycerides (TG), LDL, and very low-density lipoprotein (VLDL), while showing a positive association with HDL. These findings underscore the significant role of adiponectin in lipid metabolism, particularly its influence on HDL dynamics^64^. Despite the established inverse correlation between adiponectin levels and both overall and central adiposity, the precise role of adiponectin in the pathogenesis of insulin resistance remains incompletely elucidated^73–75^. In a cohort of Chinese patients, those with adiponectin variants rs2241766 and rs266729 exhibited an elevated risk for dyslipidemia, atherosclerosis, and coronary artery disease^65^. These findings suggest that adiponectin may serve as a viable target for strategies aimed at mitigating cardiovascular risk^65^.

Ghrelin is an orexigenic hormone that increases appetite by metabolic and non-homeostatic feeding, affecting the modulation of reward, memory and motivated feeding behavior^76^. Plasma ghrelin levels are influenced by various factors, exhibiting a postprandial decrease. In emotional eaters, ghrelin levels may be attenuated alongside reduced food intake. Notably, there is an inverse correlation between ghrelin levels and body weight, with obese individuals typically displaying lower ghrelin concentrations compared to their lean counterparts. Furthermore, elevated ghrelin levels are often associated with reduced caloric intake and conditions such as cachexia^77^. The lack of a significant difference in ghrelin levels observed in this study may be attributed to the limited sample size and dietary factors. Specifically, diet induced obesity DIO impacts the expression of the neuroendocrine ghrelin system, and it has been established that weight loss can reverse ghrelin resistance^76^.

This study has several strengths. It is one of the few to investigate FTO and MC4R gene expression in gastric tissue from humans and to correlate this with systemic hormonal and metabolic parameters. The case–control design included tightly phenotyped participants, and inverse propensity score weighting was applied to reduce confounding. The integration of qRT-PCR, ELISA, and metabolic markers in the same cohort provides a multi-dimensional view of obesity-related dysregulation.

However, several limitations must be acknowledged. The cross-sectional design precludes assessment of causality or temporal changes. In particular, the observed positive association between gastric MC4R expression and HOMA-IR should be interpreted as correlational rather than causal. Because HOMA-IR is modulated by multiple concurrent metabolic influences, our cross-sectional design cannot delineate directional or mechanistic pathways underlying this relationship. Genotyping and methylation analyses were not performed, limiting mechanistic insight into SNP–expression–phenotype pathways. The use of a single housekeeping gene, GAPDH, may also be considered a limitation; however, its stability was verified across all samples, and prior gastric expression studies have confirmed its suitability for normalization. Although RNA extraction was standardized to the mucosal layer in both groups, surgical (full-thickness) and endoscopic (mucosal) sampling methods inherently differ, which may introduce minor bias related to tissue handling or residual submucosal contamination. Ghrelin isoforms were not separately measured, and the sample size may have limited power to detect subtle associations. Finally, while control subjects were rigorously screened, biopsies from the fundus may not fully reflect global gastric or intestinal expression patterns. Additionally, because qRT-PCR was performed on bulk gastric tissue, the analysis cannot determine the precise cellular source of MC4R expression, which may arise from endocrine or neuronal elements within the gastric wall.

Future studies should investigate the longitudinal behavior of gastric FTO and MC4R expression following interventions such as metabolic surgery or caloric restriction. Parallel genotyping and methylation profiling could help elucidate whether expression is primarily genetic or environmentally driven. Functional studies using organoids or animal models could explore whether modulating gastric MC4R expression improves insulin sensitivity or adipokine balance. Expanding such analyses to multi-ethnic populations will further clarify the generalizability and clinical significance of peripheral gene regulation in obesity.

## Conclusion

In conclusion, this study demonstrates altered expression of FTO and MC4R in the gastric tissue of patients with clinical obesity and suggests links with adipokine regulation and insulin resistance. These findings support the role of peripheral tissues in obesity pathophysiology and highlight gastric molecular profiling as a promising area for future metabolic research.

## Data Availability

The datasets generated and/or analyzed during the current study are available from the corresponding author on reasonable request. The data are stored in controlled-access storage at the Department of Biomedical Informatics and Medical Statistics, Medical Research Institute.

## Abbreviations

AUC: Area under the curve
BMI: Body mass index
cDNA: Complementary DNA
CI: Confidence interval
ELISA: Enzyme-linked immunosorbent assay
FBG: Fasting blood glucose
FTO: Fat mass and obesity-associated gene
GAPDH: Glyceraldehyde 3-phosphate dehydrogenase
HDL: High-density lipoprotein
HOMA-IR: Homeostatic model assessment for insulin resistance
IPSW: Inverse propensity score weighting
LDL: Low-density lipoprotein
LNA: Locked nucleic acid
MC4R: Melanocortin 4 receptor
qRT-PCR: Quantitative real-time polymerase chain reaction
SD: Standard deviation
SE: Standard error
SG: Sleeve gastrectomy
SNP: Single nucleotide polymorphism
TWANG: Toolkit for weighting and analysis of nonequivalent groups
WC: Waist circumference
WHR: Waist-to-hip ratio

## References

[1] Antouri, Z. et al. The impact of obesity on chronic diseases: type 2 diabetes, heart disease, and high blood pressure. *Appl. Math. Sci. Eng.* **32**, 2422061. 10.1080/27690911.2024.2422061 (2024).

[2] Masood, B. & Moorthy, M. Causes of obesity: a review. *Clin. Med. (Lond)*. **23**, 284–291. 10.7861/clinmed.2023-0168 (2023). 37524429 10.7861/clinmed.2023-0168 PMC10541056

[3] Loos, R. J. F. & Yeo, G. S. H. The genetics of obesity: from discovery to biology. *Nat. Rev. Genet.* **23**, 120–133. 10.1038/s41576-021-00414-z (2022). 34556834 10.1038/s41576-021-00414-z PMC8459824

[4] Tarasconi, A. et al. Perforated and bleeding peptic ulcer: WSES guidelines. *World J. Emerg. Surg.* **15**10.1186/s13017-019-0283-9 (2020). 10.1186/s13017-019-0283-9 PMC6947898 31921329

[5] Appleton, J. The Gut-Brain axis: influence of microbiota on mood and mental health. *Integr. Med. (Encinitas)*. **17**, 28–32 (2018). 31043907 PMC6469458

[6] Clemente-Suárez, V. et al. (ed, J.) The role of adipokines in health and disease. *Biomedicines* **11**10.3390/biomedicines11051290 (2023). 10.3390/biomedicines11051290 PMC10216288 37238961

[7] Wondmkun, Y. T. & Obesity Insulin Resistance, and type 2 diabetes: associations and therapeutic implications. *Diabetes Metab. Syndr. Obes.* **13**, 3611–3616. 10.2147/dmso.S275898 (2020). 33116712 10.2147/DMSO.S275898 PMC7553667

[8] Dalamaga, M., Diakopoulos, K. N. & Mantzoros, C. S. The role of adiponectin in cancer: a review of current evidence. *Endocr. Rev.* **33**, 547–594. 10.1210/er.2011-1015 (2012). 22547160 10.1210/er.2011-1015 PMC3410224

[9] Fatima, M. T., Ahmed, I., Fakhro, K. A. & Akil, A. S. A. Melanocortin-4 receptor complexity in energy homeostasis,obesity and drug development strategies. *Diabetes Obes. Metab.* **24**, 583–598. 10.1111/dom.14618 (2022). 34882941 10.1111/dom.14618 PMC9302617

[10] Nguyen, T. M. D. & Adiponectin Role in physiology and pathophysiology. *Int. J. Prev. Med.* **11**, 136. 10.4103/ijpvm.IJPVM_193_20 (2020). 33088464 10.4103/ijpvm.IJPVM_193_20 PMC7554603

[11] Ibrahim Abdalla, M. M. Ghrelin - Physiological functions and regulation. *Eur. Endocrinol.* **11**, 90–95. 10.17925/ee.2015.11.02.90 (2015). 29632576 10.17925/EE.2015.11.02.90 PMC5819073

[12] Mahmoud, R., Kimonis, V. & Butler, M. G. Genetics of obesity in humans: A clinical review. *Int. J. Mol. Sci.* **23**10.3390/ijms231911005 (2022). 10.3390/ijms231911005 PMC9569701 36232301

[13] Sanghera, D. K., Bejar, C., Sharma, S., Gupta, R. & Blackett, P. R. Obesity genetics and cardiometabolic health: potential for risk prediction. *Diabetes Obes. Metab.* **21**, 1088–1100. 10.1111/dom.13641 (2019). 30667137 10.1111/dom.13641 PMC6530772

[14] Chami, N., Preuss, M., Walker, R. W., Moscati, A. & Loos, R. J. F. The role of polygenic susceptibility to obesity among carriers of pathogenic mutations in MC4R in the UK biobank population. *PLoS Med.* **17**, e1003196. 10.1371/journal.pmed.1003196 (2020). 32692746 10.1371/journal.pmed.1003196 PMC7373259

[15] Xi, B. et al. Common polymorphism near the MC4R gene is associated with type 2 diabetes: data from a meta-analysis of 123,373 individuals. *Diabetologia* **55**, 2660–2666. 10.1007/s00125-012-2655-5 (2012). 22869321 10.1007/s00125-012-2655-5

[16] Koochakpoor, G., Hosseini-Esfahani, F., Daneshpour, M. S., Hosseini, S. A. & Mirmiran, P. Effect of interactions of polymorphisms in the Melanocortin-4 receptor gene with dietary factors on the risk of obesity and type 2 diabetes: a systematic review. *Diabet. Med.* **33**, 1026–1034. 10.1111/dme.13052 (2016). 26666384 10.1111/dme.13052

[17] Adamska-Patruno, E. et al. An association between diet and MC4R genetic Polymorphism, in relation to obesity and metabolic Parameters-A cross sectional Population-Based study. *Int. J. Mol. Sci.* **22**10.3390/ijms222112044 (2021). 10.3390/ijms222112044 PMC8584592 34769477

[18] Eisenberg, D. et al. American Society of Metabolic and Bariatric Surgery (ASMBS) and International Federation for the Surgery of Obesity and Metabolic Disorders (IFSO) Indications for Metabolic and Bariatric Surgery. *Obes Surg* 33, 3–14, (2022). 10.1007/s11695-022-06332-1 (2023). 10.1007/s11695-022-06332-1 PMC9834364 36336720

[19] Rubino, F. et al. Definition and diagnostic criteria of clinical obesity. *Lancet Diabets Endocrionol.* **13**, 221–262. 10.1016/s2213-8587(24)00316-4 (2025). 10.1016/S2213-8587(24)00316-4 PMC11870235 39824205

[20] Garrow, J. S. & Webster, J. Quetelet’s index (W/H2) as a measure of fatness. *Int. J. Obes.* **9**, 147–153 (1985). 4030199

[21] Association, W. M. World medical association declaration of helsinki: ethical principles for medical research involving human participants. *JAMA* **333**, 71–74. 10.1001/jama.2024.21972 (2025). 39425955 10.1001/jama.2024.21972

[22] Kozera, B. & Rapacz, M. Reference genes in real-time PCR. *J. Appl. Genet.* **54**, 391–406. 10.1007/s13353-013-0173-x (2013). 24078518 10.1007/s13353-013-0173-x PMC3825189

[23] Livak, K. J. & Schmittgen, T. D. Analysis of relative gene expression data using real-time quantitative PCR and the 2(-Delta Delta C(T)) Method. *Methods* 25, 402–408, (2001). 10.1006/meth.2001.1262 10.1006/meth.2001.1262 11846609

[24] Gowayed, M. A., Achy, E., Kamel, S., El-Tahan, R. A. & M. A. & Polymyxin B prevents the development of adjuvant arthritis via modulation of TLR/Cox-2 signaling pathway. *Life Sci.* **259**, 118250. 10.1016/j.lfs.2020.118250 (2020). 32791152 10.1016/j.lfs.2020.118250

[25] Yin, D. et al. FTO: a critical role in obesity and obesity-related diseases. *Br. J. Nutr.* **130**, 1657–1664. 10.1017/S0007114523000764 (2023). 36944362 10.1017/S0007114523000764

[26] Franzago, M. et al. Fat mass and obesity-associated (FTO) gene epigenetic modifications in gestational diabetes: new insights and possible pathophysiological connections. *Acta Diabetol.* **58**, 997–1007. 10.1007/s00592-020-01668-5 (2021). 33743080 10.1007/s00592-020-01668-5 PMC8272710

[27] Czogała, W. et al. FTO and PLAG1 genes expression and FTO methylation predict changes in Circulating levels of adipokines and Gastrointestinal peptides in children. *Nutrients* **13**10.3390/nu13103585 (2021). 10.3390/nu13103585 PMC8538237 34684585

[28] Kwon, E. J. et al. Association between the DNA methylations of POMC, MC4R, and HNF4A and metabolic profiles in the blood of children aged 7–9 years. *BMC Pediatr.* **18**, 121. 10.1186/s12887-018-1104-0 (2018). 29598821 10.1186/s12887-018-1104-0 PMC5877386

[29] Mankowska, M. et al. Polymorphism and methylation of the MC4R gene in obese and non-obese dogs. *Mol. Biol. Rep.* **44**, 333–339. 10.1007/s11033-017-4114-3 (2017). 28755272 10.1007/s11033-017-4114-3 PMC5579139

[30] Kwon, E. J. et al. DNA methylations of MC4R and HNF4α are associated with increased triglyceride levels in cord blood of preterm infants. *Med. (Baltim).* **95**, e4590. 10.1097/md.0000000000004590 (2016). 10.1097/MD.0000000000004590 PMC5008556 27583872

[31] Peng, S. et al. Identification of Entacapone as a chemical inhibitor of FTO mediating metabolic regulation through FOXO1. *Sci. Transl Med.* **11**10.1126/scitranslmed.aau7116 (2019). 10.1126/scitranslmed.aau7116 30996080

[32] Huang, C., Chen, W. & Wang, X. Studies on the fat mass and obesity-associated (FTO) gene and its impact on obesity-associated diseases. *Genes Dis.* **10**, 2351–2365. 10.1016/j.gendis.2022.04.014 (2023). 37554175 10.1016/j.gendis.2022.04.014 PMC10404889

[33] Tao, Y. X. The melanocortin-4 receptor: physiology, pharmacology, and pathophysiology. *Endocr. Rev.* **31**, 506–543. 10.1210/er.2009-0037 (2010). 20190196 10.1210/er.2009-0037 PMC3365848

[34] Czajkowski, P. et al. Dietary fiber intake May influence the impact of FTO genetic variants on obesity parameters and lipid Profile-A cohort study of a Caucasian population of Polish origin. *Antioxid. (Basel)*. 10. 10.3390/antiox10111793 (2021). 10.3390/antiox10111793 PMC8614705 34829664

[35] Katus, U. et al. Association of FTO rs1421085 with obesity, diet, physical activity, and socioeconomic status: A longitudinal birth cohort study. *Nutr. Metab. Cardiovasc. Dis.* **30**, 948–959. 10.1016/j.numecd.2020.02.008 (2020). 32402589 10.1016/j.numecd.2020.02.008

[36] Hosseini-Esfahani, F. et al. Mediterranean dietary pattern adherence modify the association between FTO genetic variations and obesity phenotypes. *Nutrients* 9, (2017). 10.3390/nu9101064 10.3390/nu9101064 PMC5691681 28954439

[37] Isgin-Atici, K. et al. FTO gene–lifestyle interactions on serum adiponectin concentrations and central obesity in a Turkish population. *Int. J. Food Sci. Nutr.* **72**, 375–385. 10.1080/09637486.2020.1802580 (2021). 32746650 10.1080/09637486.2020.1802580

[38] O’Leary, N. A. et al. Reference sequence (RefSeq) database at NCBI: current status, taxonomic expansion, and functional annotation. *Nucleic Acids Res.* **44**, D733–745. 10.1093/nar/gkv1189 (2016). 26553804 10.1093/nar/gkv1189 PMC4702849

[39] Czogała, W. et al. Methylation and expression of FTO and PLAG1 genes in childhood obesity: insight into anthropometric parameters and Glucose-Lipid metabolism. *Nutrients* **13**10.3390/nu13051683 (2021). 10.3390/nu13051683 PMC8155878 34063412

[40] Saucedo, R. et al. Gene variants in the FTO gene are associated with adiponectin and TNF-alpha levels in gestational diabetes mellitus. *Diabetol. Metab. Syndr.* **9**10.1186/s13098-017-0234-0 (2017). 10.1186/s13098-017-0234-0 PMC5427601 28507607

[41] Ochoa-Rosales, C. et al. Body adiposity partially mediates the association between FTO rs9939609 and lower adiponectin levels in Chilean children. *Child. (Basel Switzerland)*. **10**10.3390/children10030426 (2023). 10.3390/children10030426 PMC10047575 36979984

[42] Guo, H. et al. Hypothalamic POMC neuron-specific knockout of MC4R affects insulin sensitivity by regulating Kir2.1. *Mol. Med. (Cambridge Mass)*. **30** (34). 10.1186/s10020-024-00804-z (2024). 10.1186/s10020-024-00804-z PMC10918880 38448811

[43] Galuppo, B. et al. Rare variants in the melanocortin 4 receptor gene (MC4R) are associated with abdominal fat and insulin resistance in youth with obesity. *Int. J. Obes. (Lond)*. **49**, 819–826. 10.1038/s41366-024-01706-0 (2025). 39738493 10.1038/s41366-024-01706-0 PMC12095050

[44] Bravard, A. et al. FTO contributes to hepatic metabolism regulation through regulation of leptin action and STAT3 signalling in liver. *Cell. Commun. Signal.* **12**10.1186/1478-811x-12-4 (2014). 10.1186/1478-811X-12-4 PMC3896784 24410832

[45] Wei, D. et al. Leptin reduces Plin5 m(6)A methylation through FTO to regulate lipolysis in piglets. *Int. J. Mol. Sci.* **22**10.3390/ijms221910610 (2021). 10.3390/ijms221910610 PMC8508756 34638947

[46] Ali, E. M. M. et al. Fat mass and obesity-associated (FTO) and leptin receptor (LEPR) gene polymorphisms in Egyptian obese subjects. *Arch. Physiol. Biochem.* **127**, 28–36. 10.1080/13813455.2019.1573841 (2021). 30767572 10.1080/13813455.2019.1573841

[47] Grunnet, L. G. et al. Regulation and function of FTO mRNA expression in human skeletal muscle and subcutaneous adipose tissue. *Diabetes* **58**, 2402–2408. 10.2337/db09-0205 (2009). 19587359 10.2337/db09-0205 PMC2750213

[48] Panaro, Brandon L.et al. The Melanocortin-4 receptor Is expressed in enteroendocrine L cells and regulates the release of Peptide YY and Glucagon-like Peptide 1 In Vivo. *Cell Metabolism*** 20**, 1018–1029, doi:10.1016/j.cmet.2014.10.004 (2014). 10.1016/j.cmet.2014.10.004 PMC4255280 25453189

[49] Wei, R., Li, D., Jia, S., Chen, Y. & Wang, J. MC4R in central and peripheral systems. *Adv. Biology*. **7**, e2300035. 10.1002/adbi.202300035 (2023). 10.1002/adbi.202300035 37043700

[50] Bayhaghi, G., Karim, Z. A. & Silva, J. Descriptive analysis of MC4R gene variants associated with obesity listed on clinvar. *Sci. Prog*. **107**, 368504241297197. 10.1177/00368504241297197 (2024). 39552559 10.1177/00368504241297197 PMC11571248

[51] Raskiliene, A., Smalinskiene, A., Kriaucioniene, V., Lesauskaite, V. & Petkeviciene, J. Associations of MC4R, LEP, and LEPR polymorphisms with Obesity-Related parameters in childhood and adulthood. *Genes (Basel)*. 12. 10.3390/genes12060949 (2021). 10.3390/genes12060949 PMC8235002 34205732

[52] Hainer, V. et al. Melanocortin pathways: suppressed and stimulated melanocortin-4 receptor (MC4R). *Physiol. Res.* **69**, S245–s254. 10.33549/physiolres.934512 (2020). 33094623 10.33549/physiolres.934512 PMC8603737

[53] Hammad, M. M. et al. MC4R variant rs17782313 associates with increased levels of DNAJC27, Ghrelin, and visfatin and correlates with obesity and hypertension in a Kuwaiti cohort. *Front. Endocrinol. (Lausanne)*. **11**, 437. 10.3389/fendo.2020.00437 (2020). 32733386 10.3389/fendo.2020.00437 PMC7358550

[54] Obradovic, M. et al. Leptin and obesity: role and clinical implication. *Front. Endocrinol. (Lausanne)*. **12**, 585887. 10.3389/fendo.2021.585887 (2021). 34084149 10.3389/fendo.2021.585887 PMC8167040

[55] Dubuc, G. R., Phinney, S. D., Stern, J. S. & Havel, P. J. Changes of serum leptin and endocrine and metabolic parameters after 7 days of energy restriction in men and women. *Metabolism* **47**, 429–434. 10.1016/s0026-0495(98)90055-5 (1998). 9550541 10.1016/s0026-0495(98)90055-5

[56] Hernández, C. et al. Influence of surgical stress and parenteral nutrition on serum leptin concentration. *Clin. Nutr.* **19**, 61–64. 10.1054/clnu.1999.0075 (2000). 10700536 10.1054/clnu.1999.0075

[57] Achari, A. E. & Jain, S. K. Adiponectin, a therapeutic target for Obesity, Diabetes, and endothelial dysfunction. *Int. J. Mol. Sci.* **18**10.3390/ijms18061321 (2017). 10.3390/ijms18061321 PMC5486142 28635626

[58] Nigro, E. et al. New insight into adiponectin role in obesity and obesity-related diseases. *Biomed Res Int* 658913, (2014). 10.1155/2014/658913 (2014). 10.1155/2014/658913 PMC4109424 25110685

[59] Luo, L. & Liu, M. Adiponectin: friend or foe in obesity and inflammation. *Med. Rev. *** 2**, 349–362,) 2, 349–362, (2021). 10.1515/mr-2022-0002 (2022). 10.1515/mr-2022-0002 PMC10388816 37724325

[60] Blüher, M. Metabolically healthy obesity. *Endocr. Rev.* **41**10.1210/endrev/bnaa004 (2020). 10.1210/endrev/bnaa004 PMC7098708 32128581

[61] Karelis, A. D. Metabolically healthy but obese individuals. *Lancet* **372**, 1281–1283. 10.1016/s0140-6736(08)61531-7 (2008). 18929889 10.1016/S0140-6736(08)61531-7

[62] Magge, S. N. et al. Adiponectin is associated with favorable lipoprotein profile, independent of BMI and insulin resistance, in adolescents. *J. Clin. Endocrinol. Metab.* **96**, 1549–1554. 10.1210/jc.2010-2364 (2011). 21367935 10.1210/jc.2010-2364 PMC3085202

[63] Akhtar, Y. et al. Relationship of serum adiponectin levels with lipid profile in diabetic and Non-Diabetic pregnant women. *Nigerian J. Basic. Clin. Sci.* **20**, 46–50. 10.4103/njbcs.njbcs_40_22 (2023).

[64] Izadi, V., Farabad, E. & Azadbakht, L. Epidemiologic evidence on serum adiponectin level and lipid profile. *Int. J. Prev. Med.* **4**, 133–140 (2013). 23543874 PMC3604843

[65] Wang, G., Wang, Y. & Luo, Z. Effect of adiponectin variant on lipid profile and plasma adiponectin levels: A multicenter systematic review and Meta-Analysis. *Cardiovasc. Ther.* **2022** (4395266). 10.1155/2022/4395266 (2022). 10.1155/2022/4395266 PMC9283072 35909951

[66] Fuster, J. J., Ouchi, N., Gokce, N. & Walsh, K. Obesity-Induced changes in adipose tissue microenvironment and their impact on cardiovascular disease. *Circul. Res.* **118**, 1786–1807. 10.1161/circresaha.115.306885 (2016). 10.1161/CIRCRESAHA.115.306885 PMC4887147 27230642

[67] Kalkman, H. O. An explanation for the adiponectin paradox. *Pharmaceuticals* **14**, 1266 (2021). 34959666 10.3390/ph14121266 PMC8703455

[68] Woodward, L., Akoumianakis, I. & Antoniades, C. Unravelling the adiponectin paradox: novel roles of adiponectin in the regulation of cardiovascular disease. *Br. J. Pharmacol.* **174**, 4007–4020. 10.1111/bph.13619 (2017). 27629236 10.1111/bph.13619 PMC5659989

[69] Aguilar-Salinas, C. A. et al. High adiponectin concentrations are associated with the metabolically healthy obese phenotype. *J. Clin. Endocrinol. Metab.* **93**, 4075–4079. 10.1210/jc.2007-2724 (2008). 18682512 10.1210/jc.2007-2724

[70] Oulhote, Y., Chevrier, J. & Bouchard, M. F. Exposure to polybrominated Diphenyl ethers (PBDEs) and hypothyroidism in Canadian women. *J. Clin. Endocrinol. Metabolism*. **101**, 590–598. 10.1210/jc.2015-2659 (2016). 10.1210/jc.2015-2659 26606679

[71] Han, Y. et al. New advances of adiponectin in regulating obesity and related metabolic syndromes. *J. Pharm. Anal.* **14**10.1016/j.jpha.2023.12.003 (2024). 10.1016/j.jpha.2023.12.003 PMC11127227 38799237

[72] Lacroix, M. et al. Lower adiponectin levels at first trimester of pregnancy are associated with increased insulin resistance and higher risk of developing gestational diabetes mellitus. *Diabetes Care*. **36**, 1577–1583. 10.2337/dc12-1731 (2013). 23300287 10.2337/dc12-1731 PMC3661817

[73] Moon, H. U., Ha, K. H., Han, S. J., Kim, H. J. & Kim, D. J. The association of adiponectin and visceral fat with insulin resistance and β-Cell dysfunction. *J. Korean Med. Sci.* **34**, e7. 10.3346/jkms.2019.34.e7 (2019). 30618514 10.3346/jkms.2019.34.e7 PMC6318440

[74] Ruan, H. & Dong, L. Q. Adiponectin signaling and function in insulin target tissues. *J. Mol. Cell. Biol.* **8**, 101–109. 10.1093/jmcb/mjw014 (2016). 26993044 10.1093/jmcb/mjw014 PMC4816150

[75] Caselli, C. Role of adiponectin system in insulin resistance. *Mol. Genet. Metab.* **113**, 155–160. 10.1016/j.ymgme.2014.09.003 (2014). 25242063 10.1016/j.ymgme.2014.09.003

[76] Jiao, Z. T. & Luo, Q. Molecular mechanisms and health benefits of ghrelin: A narrative review. *Nutrients* **14**10.3390/nu14194191 (2022). 10.3390/nu14194191 PMC9572668 36235843

[77] Zigman, J. M., Bouret, S. G. & Andrews, Z. B. Obesity impairs the action of the neuroendocrine Ghrelin system. *Trends Endocrinol. Metab.* **27**, 54–63. 10.1016/j.tem.2015.09.010 (2016). 26542050 10.1016/j.tem.2015.09.010 PMC4814209

